# Supplementary figures and images for: Metastability as a candidate neuromechanistic biomarker of schizophrenia pathology
Source: PLoS One. 2023 Mar 23;18(3):e0282707. doi: 10.1371/journal.pone.0282707 (PMC10035891; doi:10.1371/journal.pone.0282707)

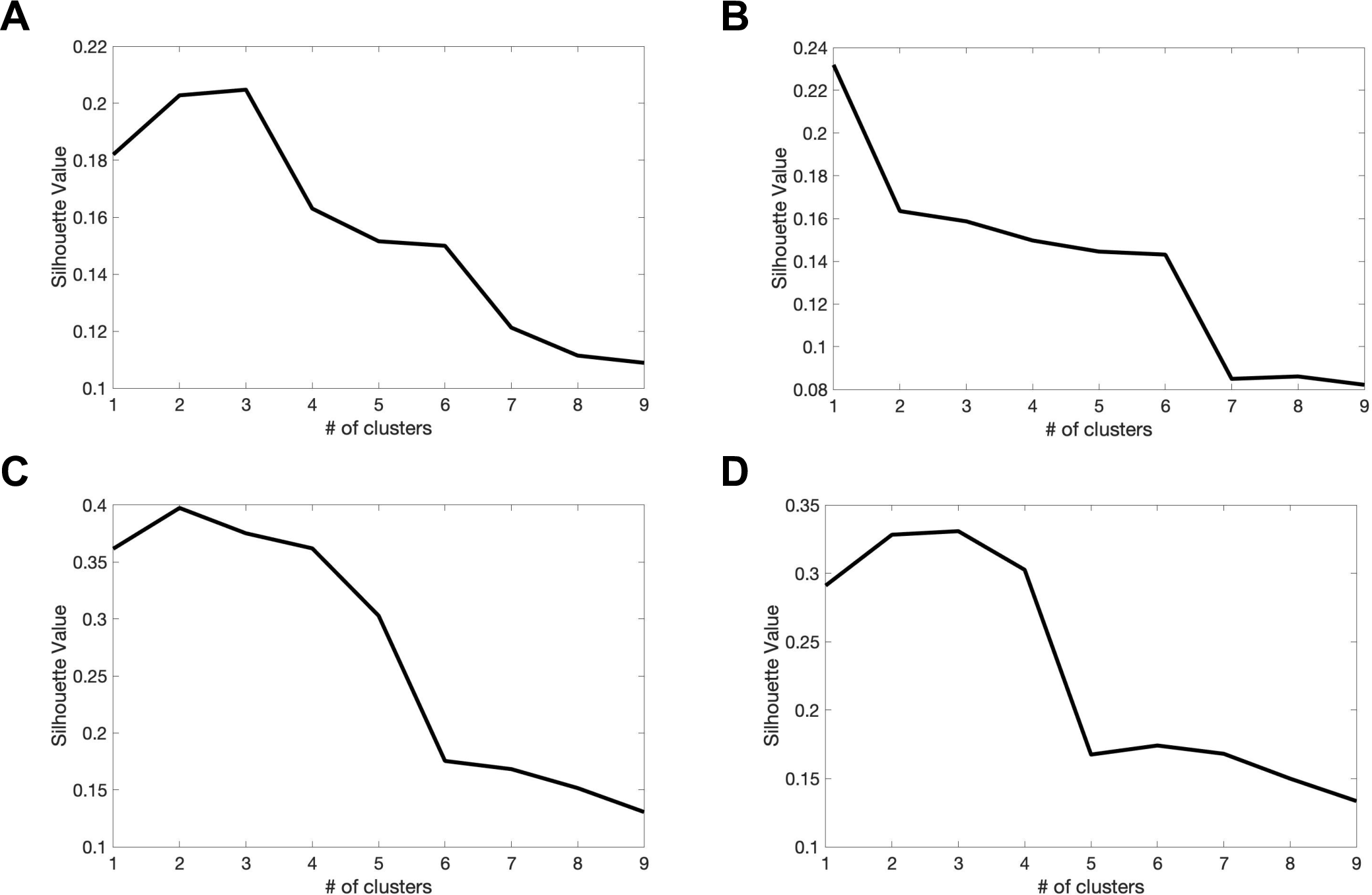

Supplement: S1 Fig — (A) HCPEP CON. (B) HCPEP NAP. (C) Cobre CON (D) Cobre SCHZ. (TIF) [file pone.0282707.s001.tif]

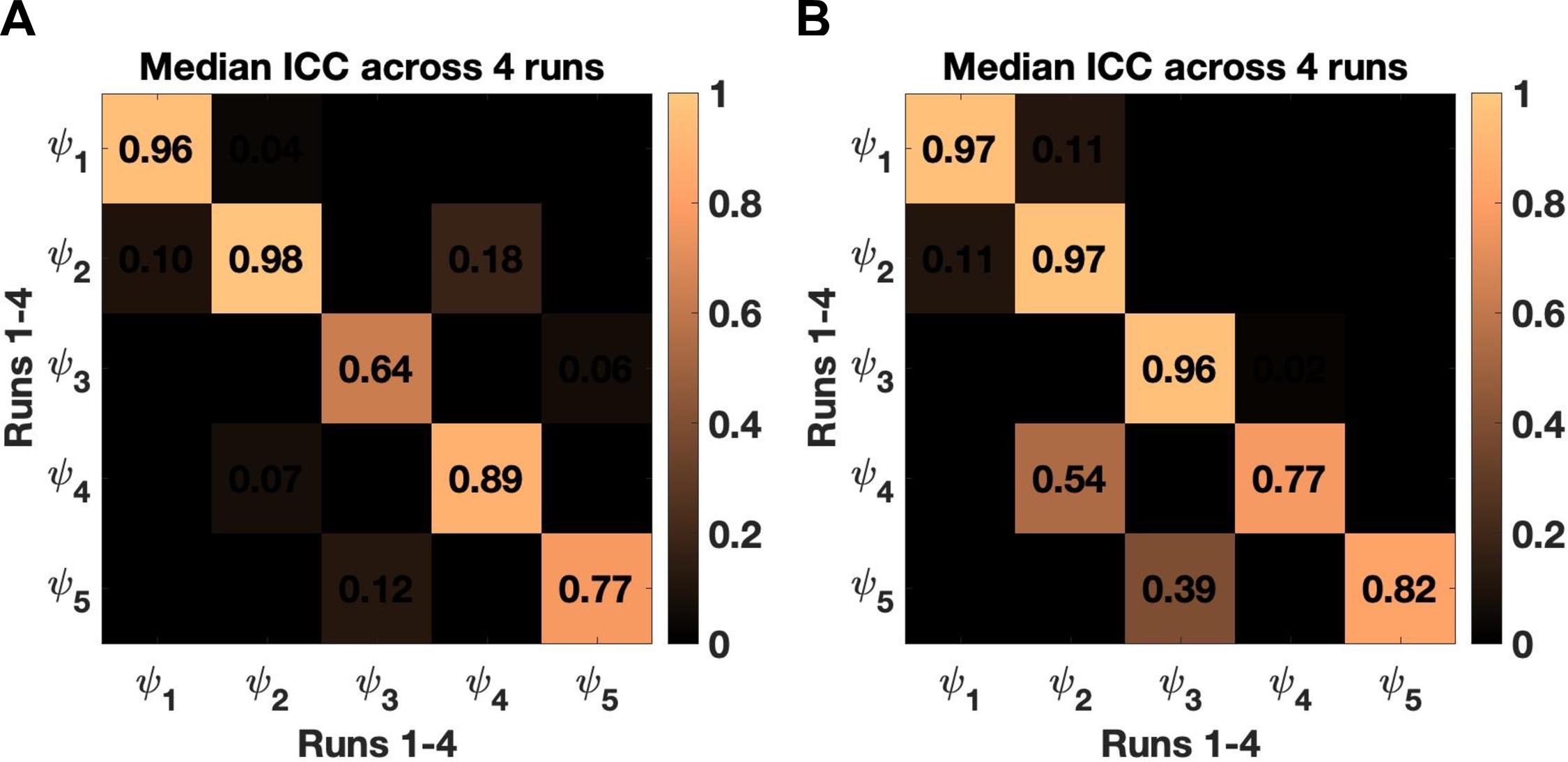

Supplement: S2 Fig — (TIF) [file pone.0282707.s002.tif]

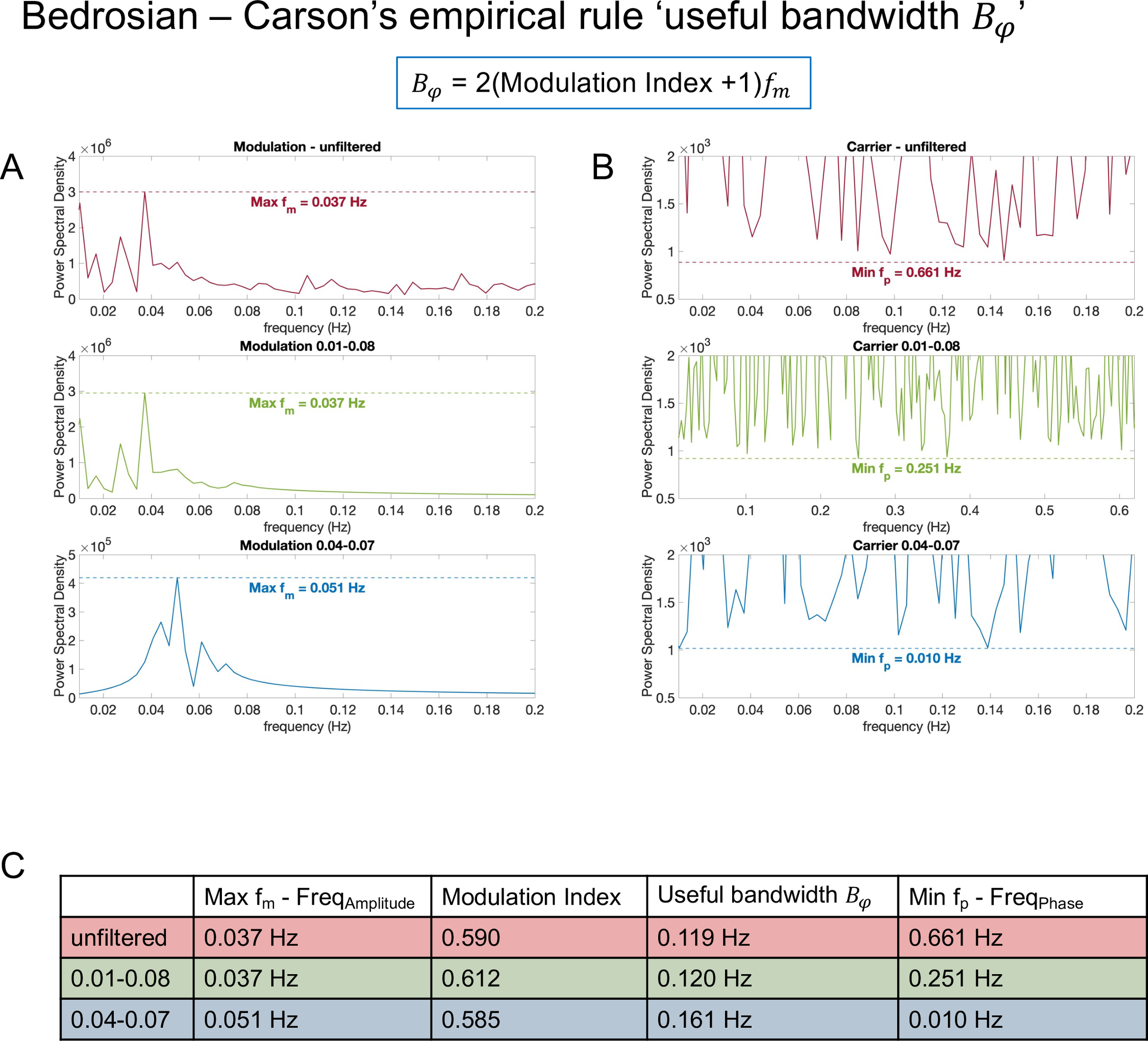

Supplement: S3 Fig — (TIF) [file pone.0282707.s003.tif]

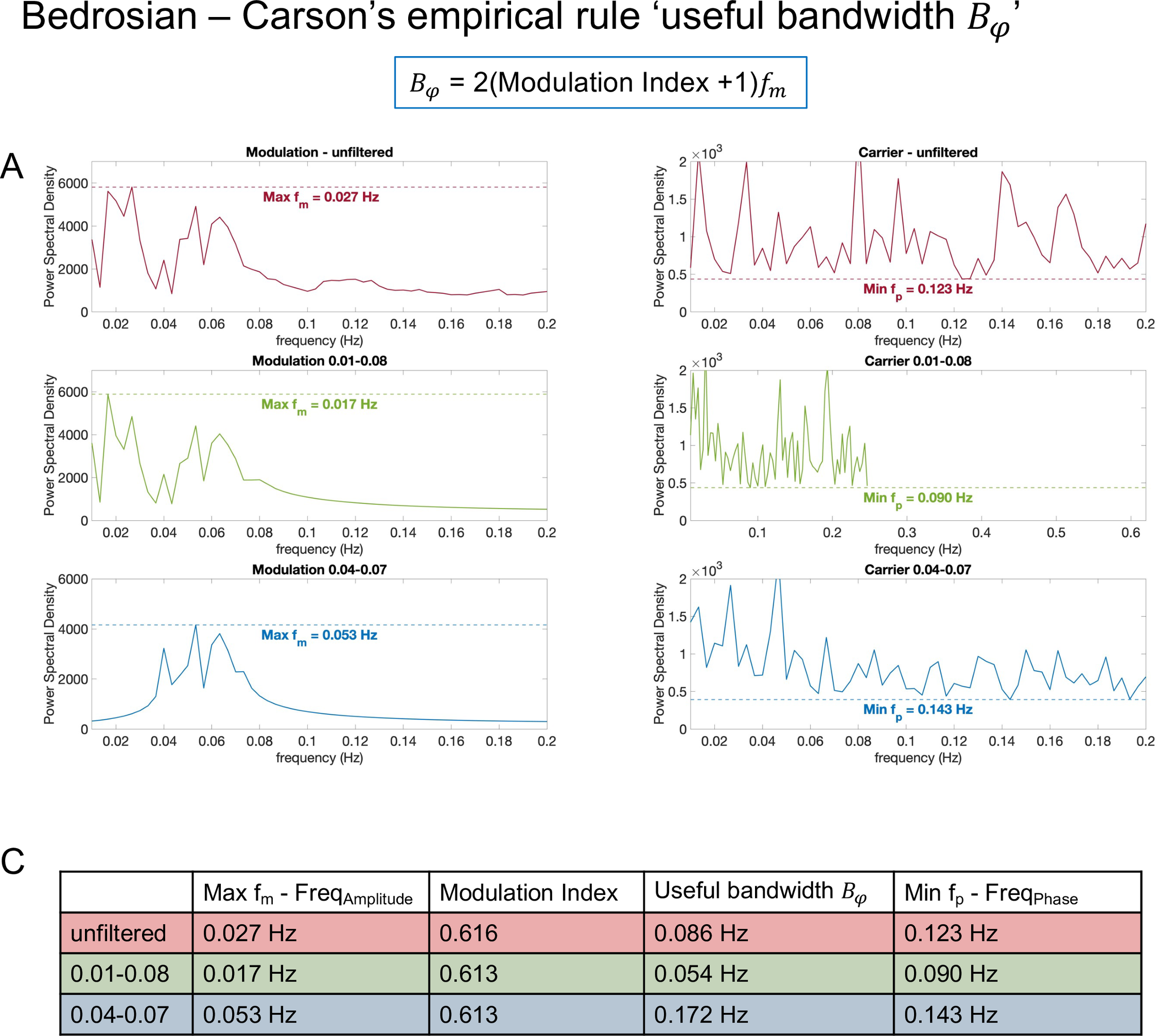

Supplement: S4 Fig — (TIF) [file pone.0282707.s004.tif]

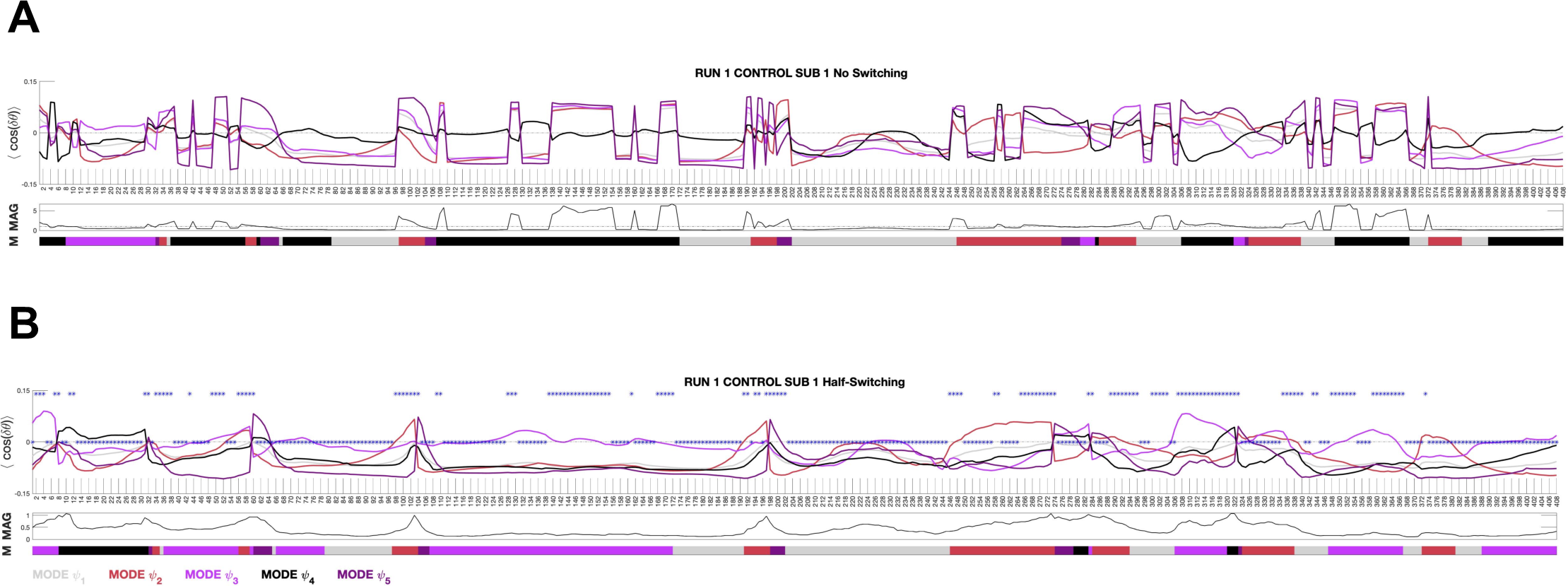

Supplement: S5 Fig — A) Time-series for the leading eigenvectors for one subject without smoothing. B) Time-series for the leading eigenvector for the same subject with half-switch smoothing. The blue asterixis indicate that half-switching occurred. (TIF) [file pone.0282707.s005.tif]
