# Supplementary material for: Metastability as a candidate neuromechanistic biomarker of schizophrenia pathology
Source: PLoS One. 2023 Mar 23;18(3):e0282707. doi: 10.1371/journal.pone.0282707 (PMC10035891; doi:10.1371/journal.pone.0282707)
Supplement: S1 Text — (PDF) [file pone.0282707.s017.pdf]

Supporting Text S1

Phase synchrony based metastability

Global and local metastability – group-level neuromechanistic biomarkers of schizophrenia

We calculated global and local metastability to compare group-level dynamics between case-control groups as the standard deviation of instantaneous phase synchrony as in [1]–[4]. Metastability measures the balance between functional integration and functional segregation, has been found to be stable across multiple acquisitions in healthy young adults [1], and is perhaps uniquely suitable for assessing schizophrenia. We calculated global and local metastability within each mode and for each run.

Global metastability

For the HCPEP dataset we first investigated group (CON, NAP), run (RUN1, RUN2, RUN3, RUN4), and group x run effects on global META. Using a 2x4 non-parametric ANOVA with the Aligned Rank Transform (ART) [5], [6], we found a significant interaction $F(3,399)=3.192$, $p=0.024$ for group x run.

Main effects of run

The main effect of run was not significant for either the CON or the NAP group.

Main effects of group

We found significant main effects of group which were driven by differences in META between CON and NAP in RUN4 ($p=0.019$, *effect size*= 0.202). See Table 1 for complete results.

Table 1. Non-parametric statistical tests for differences in global META between CON and NAP across runs.

	META					
Step 1	2x4 non-parametric ANOVA using ART					

	Term	F	Df	Df.res	Pr(>F)
COND	COND	0.235	1	133	0.629
RUN	RUN	1.302	3	399	0.273
COND:RUN	COND:RUN	3.192	3	399	0.024

Step 2 CON:1x4 repeated measures non-parametric ANOVA using Friedmann test

chi-squared	df	p.value	method	data
0.872	3	0.832	Friedman	META and RUN and SUB

Step 4 NAP:1x4 repeated measures non-parametric ANOVA using Friedmann test

chi-squared	df	p.value	method	data
6.673	3	0.083	Friedman	META and RUN and SUB

Step 6 Non-parametric Wilcox test between CON and NAP for each run

	.y.	group1	group2	n1	n2	statistic	p	p.signif	effsize	magnitude
RUN1	META	CON	NAP	53	82	2082	0.683	ns	0.035	small
RUN2	META	CON	NAP	53	82	2131	0.852	ns	0.016	small
RUN3	META	CON	NAP	53	82	2003	0.445	ns	0.066	small
RUN4	META	CON	NAP	53	82	2694	0.019	*	0.202	small

ART, Aligned Rank Transform; CON, controls; NAP, non-affective psychosis

For the Cobre dataset, we found a statistically significant difference $t(126)=-3.35$, $p=0.002$ between CON and SCHZ for global metastability.

Local Metastability

Global metastability reflects the average metastability across the modes. We were interested to also assess the local metastability in the modes.

For the HCPEP dataset we first investigated group (CON, NAP), run (RUN1, RUN2, RUN3, RUN4), and group x run effects on global META for each mode $\psi_1, \psi_2, \psi_3, \psi_4$, ψ_5). Using a 2x4 non-parametric ANOVA with the Aligned Rank Transform (ART) [5], [6], we found significant interactions for run x group in ψ_4 $F(3,399)=3.665$, $p=0.013$, ψ_5 $F(3,399)=11.325$, $p<0.001$.

Main effects of run

In the CON group, we found significant main effects of run in ψ_5, χ^2 = 9, $p<0.03$. In the NAP group, we found significant main effects of run in ψ_4, χ^2 = 19.77, $p<0.001$

and in ψ_5, for χ^2 = 21.81, *p*<0.001. The drivers for these effects and the associated effect sizes are detailed in S2 Supplementary Information.

Main effects of group

We found significant main effects of group in modes ψ_4 and ψ_5. The effect sizes of these differences were compared to the largest effect size between any pair of runs for that mode (ψ_4, *effect size*=0.308, ψ_5, *effect size*=0.410). We retained only group differences that were greater these run effects. We thus found significant group differences for ψ_5 in RUN1 (*p*<0.001, *effect size*=0.442).

We found a significant main effect of group for ψ_1, *p*=0.0361, *effect size*=0.090. There were no significant main effects of group for ψ_2 and ψ_3.

We thus inferred that mode metastability differed between CON and NAP in ψ_1 in all runs, and in ψ_5 in RUN1.

For the Cobre dataset, we found significant differences in mode META between CON and SCHZ in ψ_3 *t*(128)=-4.69, *p*<0.001, and ψ_5 *t*(122)=-3.550, *p*=0.003.

Complete statistical results for each dataset can be found in S2 Supporting Information. S6 Fig below provides an overview of mode metastability data for each dataset in the form of raincloud plots.

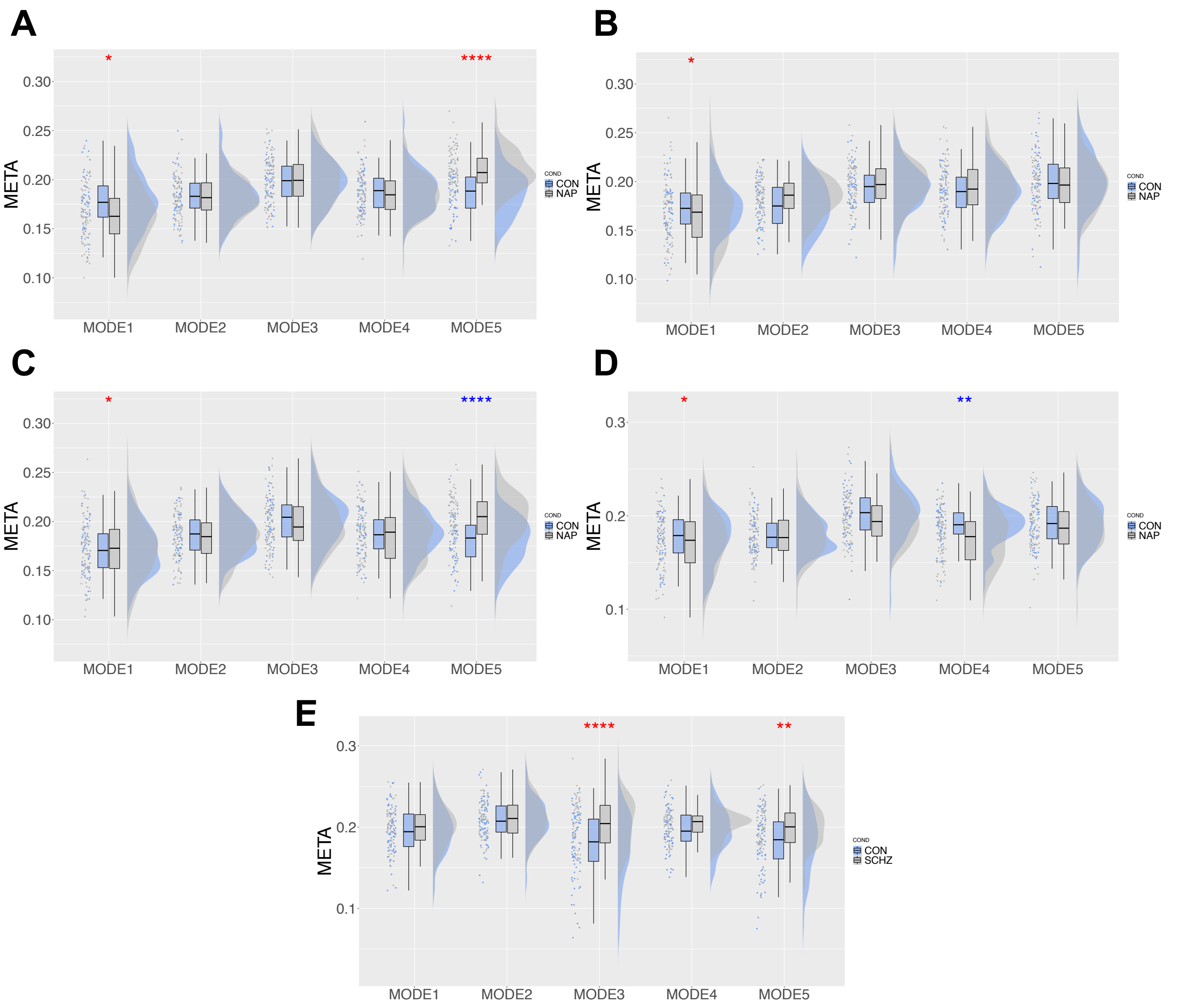

Fig S6. Raincloud plots for META in each mode for HCPEP and Cobre datasets. Raincloud plots show from left to right the raw data, boxplots showing the median, upper and lower quartiles, upper and lower extremes, and the distributions of the raw data. **A)** HCPEP RUN1. **B)** HCPEP RUN2. **C)** HCPEP RUN3. **D)** HCPCP RUN4. **E)** Cobre dataset. *=0.05, **=0.01,***=0.001,****<0.001. Red * effect size between groups greater than effect size between runs. Blue * effect size between groups less than largest effect size between runs.

References

[1] F. Hancock *et al.*, ‘Metastability, fractal scaling, and synergistic information processing: What phase relationships reveal about intrinsic brain activity’, *NeuroImage*, vol. 259, p. 119433, Oct. 2022, doi: 10.1016/j.neuroimage.2022.119433.

[2] M. Wildie and M. Shanahan, ‘Metastability and chimera states in modular delay and pulse-coupled oscillator networks’, *Chaos Interdiscip. J. Nonlinear Sci.*, vol. 22, no. 4, p. 043131, Dec. 2012, doi: 10.1063/1.4766592.

[3] M. Shanahan, ‘Metastable chimera states in community-structured oscillator networks’, *Chaos Interdiscip. J. Nonlinear Sci.*, vol. 20, no. 1, p. 013108, Mar. 2010, doi: 10.1063/1.3305451.

[4] J. Cabral, E. Hugues, O. Sporns, and G. Deco, ‘Role of local network oscillations in resting-state functional connectivity’, *NeuroImage*, vol. 57, no. 1, pp. 130–139, Jul. 2011, doi: 10.1016/j.neuroimage.2011.04.010.

[5] L. A. Elkin, M. Kay, J. J. Higgins, and J. O. Wobbrock, ‘An Aligned Rank Transform Procedure for Multifactor Contrast Tests’, in *The 34th Annual ACM Symposium on User Interface Software and Technology*, Virtual Event USA, Oct. 2021, pp. 754–768. doi: 10.1145/3472749.3474784.

[6] J. O. Wobbrock, L. Findlater, D. Gergle, and J. J. Higgins, ‘The aligned rank transform for nonparametric factorial analyses using only anova procedures’, in *Proceedings of the SIGCHI Conference on Human Factors in Computing Systems*, Vancouver BC Canada, May 2011, pp. 143–146. doi: 10.1145/1978942.1978963.
